# Evaluation of national dental curriculum in Iran using senior dental students’ feedback

**DOI:** 10.1186/s12903-023-02757-x

**Published:** 2023-01-26

**Authors:** Arvin Salmani, Hooman Keshavarz, Majid Akbari, Mohammad Javad Kharrazifard, Shabnam Varmazyari, Mohammad Reza Khami

**Affiliations:** 1 International Campus, School of Dentistry, Tehran University of Medical Sciences, Tehran, Iran; 2 Department of Community Oral Health, School of Dentistry, Mashhad University of Medical Sciences, Mashhad, Iran; 3 Restorative Dentistry Department, Dental Research Center, School of Dentistry, Mashhad University of Medical Sciences, Mashhad, Iran; 4 School of Dentistry, Tehran University of Medical Sciences, Tehran, Iran; 5 Research Center for Caries Prevention, Dentistry Research Institute, Tehran University of Medical Sciences, Tehran, Iran; 6 Department of Community Oral Health, School of Dentistry, Tehran University of Medical Sciences, Tehran, Iran

**Keywords:** Dental curriculum, Student, Evaluation, IRAN

## Abstract

**Background:**

Dental curriculums require regular revision to stay up to date in scientifical and societal fields. Senior dental students are among the main stakeholders of such curriculums. The present study investigated the opinions of Iranian senior dental students regarding the adequacy of their dentistry program and the national dental curriculum in training a competent dentist, the program’s content, and its structure.

**Methods:**

A previously designed and validated questionnaire on the opinion of senior dental students regarding curriculum adequacy was sent to a representative in each of the country’s dental schools. Before the COVID pandemic terminated data collection, a total of 16 schools (438 students) managed to respond (37%). The questionnaire asked the students to assess the adequacy of the training received in curriculum’s theoretical and practical competencies with the help of a five-point Likert scale that ranged from “Completely inadequate” to “Completely adequate”. It also questioned them on its teaching methods and intensity. SPSS software version 24 and Chi-square test served for statistical analysis.

**Results:**

In total, the study has 438 participants, 245 female and 193 male. Significant sex differences were spotted in the responses concerning both theoretical and practical training. Regarding general training adequacy, 50 (22.6%) female students and 50 male ones (30.7%), *P* = 0.08 agreed that the program was acceptable. The numbers for students of old (more than 15 years of activity) and new schools were 47 (21.7%) and 53 (31.7%), respectively (*P* = 0.03). Nearly one-third deemed the teaching methods appropriate. Regarding the duration of curriculum phases, 33 students (8.3%) believed that basic science required extension, while 108 (28.6%) and 266 (69.1%) reported such need for pre-clinical and clinical phases. The school’s years of activity emerged as significant, as 38.1% of students from new schools versus 21.7% of those from old ones deemed the extension of pre-clinical phase necessary (*P* < 0.001).

**Conclusion:**

A significant number of Iranian senior dental students found the undergraduate dental curriculum inadequate regarding competencies, content, and teaching. Further investigations will determine whether it’s the curriculum or its implementation that warrants revision.

**Supplementary Information:**

The online version contains supplementary material available at 10.1186/s12903-023-02757-x.

## Background

Assessment of undergraduate dentistry programs can be done from different perspectives. Some studies have evaluated undergraduate dental curriculums from the perspective of faculty members and dental school officials [1]. Others have considered new graduates or those with a few years of experience as the best reference point for evaluation, as they have direct exposure to the strengths and weaknesses of the curriculum through engagement in professional activities [2, 3].

Due to the rise of new sciences, the personal interests of new faculty members, demographic changes, advances in biomedical sciences, and fundamental changes in health care delivery, undergraduate dental curriculums require regular revisions [2]. The emergence of COVID-19 pandemic also impacted dental education greatly [4, 5], necessitating changes such as more emphasis on infection control, virtual learning, simulation, and virtual reality [4]. This in itself calls for regular curriculum revision and assessment.

Two valuable resources for assessment of dental education programs are the viewpoints of dental patients as the recipients of services, as well as that of health care officials as the coordinators of the health care system. However, these resources have not been properly utilized. Final year dental students are among the main stakeholders of the curriculum, and can serve as a good reference for its assessment [6–11]. Students' opinions about the content, structure, and quality of training received are essential for comprehensive evaluation of the program. They serve as an important source of information for policy makers and make the progressive quality enhancement of higher education possible [9].

In Iran, the undergraduate program of dentistry is a six-year program leading to a Doctor of Dental Surgery (DDS) degree [12]. Students directly enter dental schools after sitting the National University Entrance Exam. All state-owned and private schools are required to follow the same curriculum, developed by the Council for Dental Education in Ministry of Health and Medical Education [13]. The last major revision was undertaken in 2012. It ended up placing more emphasis on preventive dentistry, community-based education, communication skills, comprehensive care, evidence-based dentistry, and systemic disease management [14]. No other major revision has been carried out since.

Up until 2005, 18 dental schools existed in Iran, 15 state-owned and 3 private. This number witnessed a rapid growth between the years 2005 and 2013, reaching a total of 43.

Iranian dental students attend almost all courses necessary for independent practice in the first five years, getting prepared for internship and community-based education in the final year [15]. Thus, feedback from senior students can reflect the content learnt in the first five years and benefit further revisions. Expert teams comprising experienced dental professors and experts in medical education assigned by the Council for Dental Education lead these revisions [13]. From an educational point of view, progressive quality enhancement of higher education requires feedback from main stakeholders, senior dental students being one of them [16].

To the best of our knowledge, no comprehensive report is available on the opinions of these students regarding the last majorly revised version of the national dental curriculum. Therefore, the present study investigated the opinions of Iranian senior dental students regarding the adequacy of the national dental curriculum (developed and approved by the Ministry of Health) and the dentistry program (the implemented version of the curriculum within the local context of dental schools), alongside their viewpoints about the program’s content and structure. Differences that arose with respect to students’ sex, and dental schools’ years of activity were explored.

## Methods

The study protocol was approved by the Research Ethics Committee of Tehran University of Medical Sciences (TUMS) (code: IR.TUMS.DENTISTRY.REC.1397.004). TUMS and the Dental Education Excellence Center (DEEC) of Mashhad University of Medical Sciences conducted the study collaboratively. DEEC representatives, each an interested faculty member of the aforementioned schools, were designated as study coordinators. A previously designed and validated questionnaire [17] (See Additional file 1: Appendix 1) was sent to coordinators to be distributed among final year dental students in ordinary classroom settings. The study’s aims and its voluntary nature were thoroughly explained at the beginning of the questionnaire, notifying the respondent that completing it meant informed consent to participate. The participants were assured of their anonymity. They could ask for clarifications, and were free to refuse to answer any more questions and halt the process at any stage. The questionnaires, once completed, were sent to the main researcher. For the sake of data analysis, schools with more than 15 years of activity were considered old.

The data was collected in the time period between the end of first (Fall) semester and the beginning of the second (Spring) semester of the students’ final year.

In addition to age, sex, and other demographic information, the questionnaire inquired the students’ opinion about the adequacy of training, teaching methods, and program intensity.

A 47-item list of the competencies covered in the curriculum was presented to the students. They were asked to express their personal opinion regarding the adequacy of the received training in terms of a five-point Likert scale with the following options: “Completely inadequate”, “Inadequate”, “No opinion”, “Adequate”, and “Completely adequate”. They were asked to do so separately for both the theoretical and practical domains. A separate question was dedicated to assessing their overall view on the adequacy of the educational program in achieving required competencies. For the sake of data analysis, those selecting “Completely adequate” or “Adequate” were assigned to the same category [16]. Based on the distribution of the responses, and to emphasize the extreme ends of it, agreement level of less than 30% and more than 70% regarding the curriculum adequacy in achieving competencies acted as cut-off points for further analysis.

Three independent questions asked for the students’ personal opinion regarding the appropriateness of teaching methods of theoretical courses, practical courses of the pre-clinical phase, and practical courses of the clinical phase. The five-point Likert scale options were: “Completely inappropriate”, “Inappropriate”, “No opinion”, “Appropriate”, and “Completely appropriate”. For the sake of data analysis, those who selected “Completely appropriate” or “Appropriate” were assigned to the same category [16].

For each of the three phases of dental education (basic science, pre-clinical, and clinical) two additional questions were asked: one asked for the students’ personal opinion on the program intensity in that particular phase, and the other assessed their reaction to a statement about the necessity of that phase getting extended. The five-point Likert scale options for all these questions were “Completely disagree”, “Agree”, “No opinion”, “Agree”, and “Completely agree”. Again, those who opted for “Completely agree” or “Agree” were assigned to the same category [16].

### Statistical analysis

SPSS software version 24, and Chi-square test served for statistical analysis. Significance level was set at *P* < 0.05. To ensure questionnaire reliability, Cronbach’s alpha was calculated separately for questions addressing theoretical and practical adequacy.

## Results

Out of 43 Iranian dental school, 16 managed to send the completed questionnaires (response rate: 37%). In total, 438 students participated in this study, of which 245 students (55.9%) were female.

The Cronbach’s alpha for questions regarding both theoretical and practical adequacy exceeded 0.9.

Table 1 illustrates the competencies that less than 30% of the students deemed adequately covered by the dentistry program. These were mainly related to either non-clinical or clinical and complicated competencies, and some significant differences were spotted among them based on sex or the school’s working years. Statistically significant differences were found between the reponses of two sexes regarding theoretical training in “Implementation of evidence-based dentistry principles” (27.2% of males vs. 14.8% of females, *P* = 0.005), and “Maintenance of dental equipment” (22.3% of males vs. 14.8% of females, *P* = 0.046). While 29.4% of the students of new schools believed in the adequacy of theoretical training in “Complicated surgical extraction of wisdom teeth”, the corresponding figure among students in the older schools was 19.4% (*P* = 0.02). In the domain of practical training, statistically significant sex differences existed for six competencies: “Performing other intra-oral surgeries” (17.4% of males, 10.9% of females, *P* = 0.03), “Maintenance of dental equipment” (19.1% of males, 11.6% of females, *P* = 0.03), “Implementation of evidence-based dentistry principles” (26.9% of males, 12.8% of females, *P* = 0.001), “Practice management” (20.3% of males, 12.9% of females, *P* = 0.04), “Fabrication of space-maintainers” (27% of males, 16.7% of females, *P* = 0.01), and “Simple surgical extraction of wisdom teeth” (33.7% of males, 22.1% of females, *P* = 0.008). In the domain of practical training, there were statistically significant differences between the students’ responses in old and new schools with regards to: “Complicated surgical extraction of wisdom teeth” (23.5% in new schools, 10.9% in old schools, *P* = 0.001), “Performing periodontal surgeries” (23.1% in new schools, 13.7% in old schools, *P* = 0.01), “Fabrication of space-maintainers” (32.4% in new schools, 12.7% in old schools, *P* < 0.001), and “Simple surgical extraction of wisdom teeth” (39% in new schools, 18.1% in old schools, *P* < 0.001).

**Table 1:** The curriculum-defined competencies that below 30% of the Iranian senior dental students (n = 438) believed in the adequacy of dentistry program to cover them, and the distribution of the students’ responses according to their sex and dental school active years

	Total n (%)	Sex		P*	School working years		P*
		Male n (%)	Female n (%)		≤ 15 years n (%)	> 15 years n (%)	
*Theoretical*							
Implementation of evidence-based dentistry principles**	70 (20.7)	44 (27.2)	26 (14.8)	0.005	44 (23.5)	26 (17.2)	0.16
Maintenance of dental equipment	77 (18.2)	42 (22.3)	35 (14.8)	0.046	29 (15.6)	48 (20.2)	0.23
Performing other intra-oral surgeries (other than tooth extraction)	79 (18.5)	34 (17.9)	45 (19.0)	0.77	41 (21.9)	38 (15.8)	0.11
Practice management	80 (18.8)	41 (21.5)	39 (16.7)	0.21	31 (16.6)	49 (20.6)	0.29
Performing a medical research**	84 (24.7)	44 (27.2)	40 (22.5)	0.32	49 (26.2)	35 (22.9)	0.48
Endodontic re-treatment of a multiple-root tooth	88 (21.0)	45 (24.3)	43 (18.4)	0.14	39 (21.4)	49 (20.7)	0.85
Complicated surgical extraction of wisdom teeth	102 (23.8)	49 (25.8)	53 (22.2)	0.38	55 (29.4)	47 (19.4)	0.02
Performing periodontal surgeries	111 (26.1)	44 (23.3)	67 (28.4)	0.23	53 (28.5)	58 (24.3)	0.33
*Practical*							
Performing other intra-oral surgeries (other than tooth extraction)	57 (13.8)	33 (17.4)	24 (10.9)	0.03	29 (16.1)	28 (12.0)	0.23
Maintenance of dental equipment	62 (14.9)	35 (19.1)	27 (11.6)	0.03	24 (13.2)	38*16.3)	0.38
Implementation of evidence-based dentistry principles**	64 (19.5)	42 (26.9)	22 (12.8)	0.001	39 (21.5)	25 (17.0)	0.30
Practice management	67 (16.2)	37 (20.3)	30 (12.9)	0.04	28 (15.6)	39 (16.6)	0.79
Complicated surgical extraction of wisdom teeth	68 (16.3)	35 (19.1)	33 (14.1)	0.17	42 (23.5)	26 (10.9)	0.001
Performing a medical research**	68 (20.5)	35 (22.3)	33 (19.0)	0.45	36 (19.8)	32 (21.5)	0.70
Endodontic re-treatment of a multiple-root tooth	71 (17.3)	38 (20.7)	33 (14.5)	0.1	31 (17.1)	40 (17.4)	0.94
Management of medical emergencies	72 (17.5)	29 (16.1)	43 (18.6)	0.51	31 (17.3)	41 (17.7)	0.93
Performing periodontal surgeries	74 (17.8)	35 (19.1)	39 (16.7)	0.53	42 (23.1)	32 (13.7)	0.013
Fabrication of space-maintainers	89 (21.3)	50 (27.0)	39 (16.7)	0.01	59 (32.4)	30 (12.7)	< 0.001
Professional behavior with other colleagues**	93 (28.3)	44 (28.6)	49 (28.0)	0.91	49 (27.2)	44 (29.5)	0.64
Management of dental emergencies	103 (25.1)	50 (27.3)	53 (23.3)	0.36	50 (27.9)	53 (22.9)	0.25
Prescribing necessary drugs when needed	111 (27.2)	49 (27.1)	62 (27.3)	0.96	47 (26.0)	64 (28.2)	0.62
Simple surgical extraction of wisdom teeth	114 (27.2)	62 (33.7)	52 (22.1)	0.008	71 (39.0)	43 (18.1)	< 0.001

*Chi-square test

**These questions, which were at the end of the list in the distributed questionnaire, left un-answered by the students of one of the dental schools. Regarding other questions, there were up to 22 (6%) non-responses in theoretical domain, and up to 30 (7%) in practical domain

Table 2 illustrates the competencies with reportedly maximum (more than 70%) agreement among students regarding the adequacy of their teaching. These competencies were mostly related to simple clinical procedures, with some significant differences based on sex or school’s working years. While 79.4% of the male students believed in the adequacy of theoretical teaching in “Restoring a relatively small cavity”, the corresponding figure among female students was 86.9% (*P* = 0.04). Differences existed between responses of the students of new and old schools with regards to the practical teaching of these two competencies: “Pulpectomy of a deciduous molar” (78.8% in new schools, 68.8% in old schools, *P* = 0.02) and “Pulpotomy of a deciduous molar” (84.6% in new schools, 75.1% in old schools, *P* = 0.02). In the domain of practical teaching, the responses of male students differed significantly from that of female students regarding the competency of “Restoring a big cavity involving more than two surfaces of the tooth” (66.5% vs. 75.4%, *P* = 0.046). While 77.5% of students of new schools reported the practical teaching of “Pulpotomy of a deciduous molar” adequate, the corresponding figure for those who belonged to old schools was 65.3% (*P* = 0.007).

**Table 2:** The curriculum-defined competencies that more than 70% of the Iranian senior dental students (n = 438)* believed in the adequacy of dentistry program to cover them, and the distribution of the students’ responses according to their sex and dental school active years

	Total n (%)	Sex		P*	School working years		P**
		Male n (%)	Female n (%)		≤ 15 years n (%)	> 15 years n (%)	
*Theoretical*							
Interpreting intra-oral radiographs	300 (71.6)	128 (69.6)	172 (73.2)	0.41	132 (71.4)	168 (71.8)	0.92
Restoring a big cavity involving more than two surfaces of the tooth	311 (74.0)	134 (72.8)	177 (75.0)	0.61	134 (71.7)	177 (76.0)	0.32
Pulpectomy of a deciduous molar	314 (73.2)	143 (74.9)	171 (71.8)	0.48	149 (78.8)	165 (68.8)	0.02
Taking medical history	315 (73.4)	130 (68.8)	185 (77.1)	0.05	141 (75.4)	174 (71.9)	0.42
Taking dental history	325 (74.2)	136 (72.3)	189 (79.4)	0.09	142 (77.2)	183 (75.6)	0.71
Extraction of a single-root tooth	327 (77.1)	141 (75.0)	186 (78.8)	0.35	145 (78.4)	182 (76.2)	0.59
Pulpotomy of a deciduous molar	340 (79.3)	150 (78.9)	190 (79.5)	0.89	159 (84.6)	181 (75.1)	0.02
Prescribing necessary intra-oral radiographs	346 (82.0)	151 (81.6)	195 (82.3)	0.86	159 (85.9)	187 (78.9)	0.06
Endodontic treatment of a single-root tooth	346 (82.0)	151 (80.3)	195 (83.3)	0.42	151 (81.6)	195 (82.3)	0.86
Restoring deciduous teeth	349 (81.4)	153 (80.1)	196 (82.4)	0.55	157 (83.5)	192 (79.7)	0.31
Restoring a relatively small cavity	355 (83.5)	150 (79.4)	205 (86.9)	0.04	160 (85.1)	195 (82.3)	0.44
*Practical*							
Restoring a big cavity involving more than two surfaces of the tooth	293 (71.5)	121 (66.5)	172 (75.4)	0.046	125 (68.3)	168 (74.0)	0.20
Pulpotomy of a deciduous molar	295 (70.6)	127 (69.0)	168 (71.8)	0.54	141 (77.5)	154 (65.3)	0.007
E extraction of a single-root tooth	301 (73.1)	127 (70.2)	174 (75.3)	0.24	129 (71.7)	172 (74.1)	0.58
Prescribing necessary intra-oral radiographs	318 (77.4)	137 (74.9)	181 (79.4)	0.28	149 (81.9)	169 (73.8)	0.05
Endodontic treatment of a single-root tooth	321 (77.5)	140 (76.5)	181 (78.4)	0.65	145 (80.6)	176 (75.2)	0.2
Restoring deciduous teeth	325 (77.8)	136 (73.9)	189 (80.8)	0.09	145 (79.2)	180 (76.6)	0.52
Restoring a relatively small cavity	327 (79.6)	138 (75.4)	189 (82.9)	0.06	142 (78.9)	185 (80.1)	0.77

*There were up to 22 (6%) non-responses in theoretical domain, and up to 30 (7%) in practical domain

**Chi-square test

One hundred students (26.0%), 50 males and 50 females agreed or completely agreed that in general, their educational program has been adequate in achieving required competencies for a dentist (30.7% of males, 22.6% of females, *P* = 0.08). Of students in older schools, 47 (21.7%) of students, and of those in the new schools, 53 (31.7) of students, agreed with this statement (*P* = 0.03).

Check Additional file 2: Appendix 2 for the complete results of this section. For the distribution of the response to this section, including the “no opinion” option, see Additional file 3: Appendix 3, Tables S1 and S2.

Nearly one-third of students believed in the appropriateness of teaching methods in theoretical courses (118, 34.6%), practical courses of the pre-clinical phase (128, 33.7%), and practical courses of the clinical phase (109, 29.7%). No significant difference was identified with regards to sex or working years of the dental school (Table 3). For the distribution of the responses to this section, including the “no opinion” option, see Additional file 3: Appendix 3, Table S3.

**Table 3:** The proportion of the Iranian senior dental students (n = 438) who believed that the teaching methods used in the three phases of the national dental curriculum have been appropriate or completely appropriate, and the distribution of responses by sex and dental schools active years

	Total n (%)	Sex		P*	School working years		P*
		Male n (%)	Female n (%)		≤ 15 years n (%)	> 15 years n (%)	
Theoretical courses	118 (34.6)	46 (31.7)	72 (36.7)	0.336	47 (32.4)	71 (36.2)	0.47
Practical courses in the pre-clinical phase	128 (33.7)	54 (32.7)	74 (34.4)	0.730	45 (28.5)	83 (37.4)	0.07
Practical courses in the clinical phase	109 (29.7)	53 (33.8)	56 (26.7)	0.141	48 (30.6)	61 (29.0)	0.75

*Chi-square test

As Table 4 depicts, 118 male students (76.1%) and 120 female students (58.3%) deemed their program intense in the basic science phase (*P* < 0.001). The corresponding figure for students of new and old schools were 111 (72.5%) and 127 (61.1%), respectively (*P* = 0.02). Moreover, 192 students (55%) viewed the pre-clinical phase as intense, and 184 (52.4%) held the same view about the clinical phase. No significant difference with regards to sex or the school’s working years was identified (Table 4). For the distribution of the responses to this section, including the “no opinion” option, see Additional file 3: Appendix 3, Table S4.

**Table 4:** The proportion of the Iranian senior dental students (n = 438) who agreed or completely agreed with the intensity of the national dental curriculum in its three phases, and the distribution of responses by sex and dental schools’ active years

	Total n (%)	Sex		P*	School working years		P*
		Male n (%)	Female n (%)		≤ 15 years n (%)	> 15 years n (%)	
Basic science phase	238 (65.9)	118 (76.1)	120 (58.3)	< 0.001	111 (72.5)	127 (61.1)	0.02
Pre-clinical phase	192 (55.5)	87 (61.3)	105 (51.5)	0.071	89 (59.7)	103 (52.3)	0.17
Clinical phase	184 (52.4)	87 (56.5)	97 (49.2)	0.177	76 (53.5)	108 (51.7)	0.73

*Chi-square test

While 33 students (8.3%) reported the extension of the basic science phase necessary, 108 (28.6%) held the same view about the pre-clinical phase, and 266 (69.1%) about the clinical phase. The only significant difference concerned the schools’ working years, since 38.1% of the students of new schools versus 21.7% of the students of old schools believed that the pre-clinical phase requires extension (*P* < 0.001) (Table 5). For distribution of the responses to this section, including “no opinion” alternative, see Additional file 3: Appendix 3, Table S5.

**Table 5:** The proportion of the Iranian senior dental students (n = 438) who agreed or completely agreed with the necessity of the extension of the three phases of national dental curriculum, and the distribution of responses by sex and dental schools’ active years

	Total n (%)	Sex		P*	School working years		P*
		Male n (%)	Female n (%)		≤ 15 years n (%)	> 15 years n (%)	
Basic science phase	33 (8.3)	19 (10.7)	14 (6.3)	0.118	16 (9.5)	17 (7.4)	0.44
Pre-clinical phase	108 (28.6)	47 (30.1)	61 (27.6)	0.593	61 (38.1)	47 (21.7)	< 0.001
Clinical phase	266 (69.1)	108 (64.3)	158 (72.8)	0.07	107 (65.2)	159 (71.9)	0.16

*Chi-square test

## Discussion

The present study investigated the opinions of Iranian final-year dental students regarding their dental program. In our study, only about a quarter of the participants reported the adequacy of the training they received in their program acceptable, and approximately one-third believed that the teaching methods were either appropriate or completely appropriate.

The 2012 revision of the Iranian national dental curriculum tried to address the problems of the previous version such as overcrowding, lack of elective courses, inadequate emphasis on meta-competencies (such as communication skills, professionalism, evidence-based dentistry, etc.) and preventive dentistry, absence of modern educational methods, not being in accordance with community needs, and the program’s requirement-based- and not competency-based-nature [14]. As a study conducted in 2011 with the same questionnaire reported similar results [17], it can be deducted that the latest revision has been unsuccessful in increasing students’ satisfaction. However, we should take into consideration that in the 2011 study, all participating dental schools were of at least 15 years of working age while in the present study, only six schools had such background. Although the number of schools increased dramatically between 2005 and 2013, they received insufficient supply in terms of infrastructure, more specifically faculty members [18]. This led to the new schools turning to employing temporary faculty members that mostly intended to fulfill their compulsory service to compensate for their free postgraduate education. The older schools, on the other hand, continuously benefitted from the presence of permanently employed, experienced faculty members. Most of these old schools already have established postgraduate residency programs in various disciplines, while the new schools lack such luxury. Residency programs independently affect undergraduate training. While they can improve the quality of education in a particular department, they might as well lead to the constant referral of complex cases to residents and reduce the quality of the undergraduate program.

In the present study, the students’ judgement was based on their personal beliefs and opinions which comes with its own limitations. For example, it may differ from that of the other stakeholders. A 2020 study by Abdelsalam et al. that investigated the levels of satisfaction of students and faculty members with the dental curriculum at a Saudi Arabia dental school reported that faculty members had a higher level of satisfaction with the curriculum compared to students. The faculty reportedly deemed the curriculum more capable of preparing competent graduates [16].

The present findings clarify the need for the implementation of new medical education techniques. These techniques include but are not limited to: problem-based learning [19, 20], flipped classroom [21], simulation [22], virtual reality and technology-enhanced learning [23], virtual and online education [4], and gamification [24]. COVID-19 pandemic has facilitated the emergence and enhancement of some of these methods [4, 25, 26]. Skills that belong to the domains of problem solving, self-study, and lifelong learning require improvement. Teaching the skills of evidence-based dentistry and integrating them into the curriculum as a major component will help students develop them. This is even more critical when we consider that in the present study, evidence-based dentistry was among the fields with the lowest levels of reported adequacy.

The competencies with the minimum reported adequacy in both theoretical and practical domains were nearly alike. They can be divided into three categories. First, the inadequacy of the dentistry program in competencies like “Implementation of evidence-based dentistry principles” and “Performing medical research”, which might be a reflection of the shortcomings of the experts of these fields. This concern has been raised previously in a study carried out on the knowledge and attitude of Iranian dentistry faculty members towards evidence-based dentistry [27]. Second, regarding procedure-based competencies like “Performing other intra-oral surgeries (other than tooth extraction)”, “Endodontic re-treatment of a multiple-root tooth”, “Surgical extraction of wisdom teeth”, “Performing periodontal surgeries”, and “Fabrication of space-maintainers”, the inadequacy may stem from the complicated nature of the procedure. In line with these findings, a previous study reported that oral and maxillofacial surgery and endodontics were the main fields of practice among less than 20% of Iranian dentists [14]. Scarcity of referred cases with these problems to clinics of dental school might be another reason for such findings. Finally, other competencies such as “Maintenance of dental equipment”, “Practice management”, “Management of medical emergencies”, “Professional behavior with other colleagues”, and “Prescribing necessary drugs when needed” which are mainly related to soft skills, may have not received due attention from the curriculum. This concern was raised earlier: a previous study reported that the curriculum’s inadequacy was more evident in non-clinical domains compared to clinical ones [14]. Consequently, the latest revision of the curriculum put more emphasis on these competencies [14]; for instance, the courses of “Communication skills” and “Ethics and Professionalism” got expanded and underwent major transformation. Our findings indicate that there is still room for such improvements.

The findings of the present study are partly in line with research conducted in other countries. In UK, Downer et al. reported that a high percentage of students did not consider themselves prepared to carry out procedures like apicoectomies, incisional biopsies, and periodontal surgeries. The students also reported lack of awareness in the diagnosis and treatment of different emergency situations. Their study also demonstrated that the students were best prepared for tasks like periapical radiographies, permanent first molars extraction, and third molar surgeries [28]. In another study by Ryding and Murphy in Canada, both groups of former and new curriculum alumni believed that the undergraduate dentistry curriculum was sufficient in preparing them for clinical work. They, however, reported inadequate readiness when it came to tasks like performing and interpreting clinical biological tests, management of medical emergencies, assessment of growth and developmental cases and correctly referring them, and orthodontic treatments. On the other hand, they declared that they were highly prepared for single-tooth restorations, accurate and complete recording of patients’ information, selection of appropriate radiographs for diagnosis, evaluation of patients' periodontal status, and provision of initial periodontal treatments [2].

In our study, significant sex differences were generally evident between the responses, with male students finding the overall adequacy of the curriculum more satisfactory. Understanding the reason behind this disparity requires further investigation.

All the differences that emerged with regards to the schools’ working years included the students of new schools deeming the program more adequate regarding some mainly procedural competencies. The surgical extraction of wisdom teeth, periodontal surgery, and fabrication of space-maintainers, to name a few. These differences can be attributed to the presence of postgraduate students in old dental schools and the possible referral of complex cases to them.

Although approximately 65% of the students viewed the basic science phase as intense, less than 10% agreed with its extension. This could be due to Iranian dental students’ inability in establishing a clear connection between basic science and the clinical education later received. Calling attention to better teaching of basic science has been a pillar of the dental education revisions throughout the world [29]. This calls for inclusion of more integrative content in the curriculum. This finding, also, has been a common of the themes of dental curriculum revisions [30]. On the other hand, around 70% of the students agreed with the necessity of extending the clinical phase. This is in line with another finding of our study, that three quarters of students find the dentistry program incapable of preparing them for general dental practice.

A questionnaire seems to be the best practical way to obtain students' opinions regarding these issues. Still, such questionnaires have some shortcomings of their own. One such shortcoming is respondents’ lack of willingness to complete them. We strived to overcome this by stressing the importance of the students’ responses, emphasizing that they have the ability to positively impact the future curriculum revisions. The self-reported nature of these questionnaires also raises the possibility of social desirability bias [31]. Moreover, the students’ perception of confidence regarding some competencies may have been affected by the fact that they completed the questionnaire in the middle of their final year, and not at its end. It should also be noted that the students' opinions are only one source of information, and the opinions of professors, dentists, and other stakeholders needs to be obtained if an accurate picture of the shortcomings of the program is to be painted. One should be cognizant of the fact that the participating schools were mostly newly established ones. These schools tend to face challenges with the recruitment of workforce and the provision of infrastructure, amenities, and equipment. This factor may very well contribute to insufficient curriculum implementation. Since the required infrastructure, including academic staff members, did not evolve sufficiently during the 2005–2013 surge in the number of schools, there is reason for concern over the quality of education these new schools offer. It’s worth bearing in mind that none of the pioneer dental schools of Iran, i.e. those with a working age that exceeds 50 years, were a part of this study.

The results of the present study help with the planning of further revisions of the national dental curriculum in Iran. They might also come in handy for those involved in dental curriculum planning and assessment. This study has limitations such as relying on the students’ personal opinion, mostly exploring new dental schools, and being cross-sectional. Data collection proved challenging, as once completed questionnaires of 16 schools were received, COVID-19 pandemic occurred. All dental schools were closed down, and it became impossible to continue the process. Due to great post-pandemic transformations in the field of education, we found it unreasonable to resume data collection after the schools re-opened. Thus, the results require cautious interpretation. Despite this, the response rate of each participating school was relatively high, leading to data being obtained from 438 senior dental students. Although the results may not be generalizable to all dental schools, they have some implications for the newly established ones that the majority of the respondents belonged to. Moreover, the validity of the questionnaire was further confirmed by the data of the present study, which can be considered a point of strength.

## Conclusion

Overall, the undergraduate dental program is deemed inadequate from the viewpoint of Iranian senior dental students with regards to its ability to develop competencies, its content, and teaching methods. These findings call for further investigations into the national dental curriculum and the way of its implementation. A post-pandemic version of this study can prove useful and provide insight into the impact of the pandemic on dental education in Iran. Gathering information from faculty members using the same questionnaire will also shed light on the issue.

## Supplementary Information

**Additional file 1: Appendix 1.** Questionnaire.

**Additional file 2: Appendix 2.** Frequency distribution of the Iranian senior dental students (n=438) who believed in the adequacy of dentistry program to cover each of the curriculum-defined competencies, according to their gender.

**Additional file 3: Appendix 3.** Frequency distribution of the responses by Iranian senior dental students (n=438) to the questions regarding adequacy of their dentistry program to cover each of the curriculum-defined competencies, appropriateness of the teaching methods used in the three phases of the national dental curriculum, intensity of the national dental curriculum in its three phases, and agreement with the necessity of the extension of the three phases.

## Data Availability

The data supporting the findings of this study are available from TUMS school of dentistry, but restrictions apply to the availability of these data, which were used under license for the current study, and so are not publicly available. Data are however available from the authors upon reasonable request and with permission from TUMS school of dentistry.

## Abbreviations

TUMS: Tehran University of Medical Sciences
DEEC: Dental Education Excellence Center
ADEA: American Dental Education Association
DDS: Doctor of Dental Surgery
