# Supplementary material for: Evaluation of national dental curriculum in Iran using senior dental students’ feedback
Source: BMC Oral Health. 2023 Jan 26;23:45. doi: 10.1186/s12903-023-02757-x (PMC9876750; doi:10.1186/s12903-023-02757-x)
Supplement: Supplementary file 1 — Additional file 1: Appendix 1. Questionnaire. [file 12903_2023_2757_MOESM1_ESM.docx]

Section 1: Personal information of the respondent

Gender:

1. Male 2. Female

Semester:

1. 10 2.11 3.12

Section 2: Questions regarding the content of the undergraduate dental curriculum

A: In this section you face a more general question. "What is your opinion about the adequacy of your training for achievement of each of the mentioned competency?"

You are required to choose one option for each question provided. Make sure to choose options separately for theoretical and practical subjects. Stick to one option only.

|  | Theory | | | | | Practice | | | | |
| --- | --- | --- | --- | --- | --- | --- | --- | --- | --- | --- |
|  | Completely  inadequate | Inadequate | No opinion | Adequate | Completely adequate | Completely  inadequate | Inadequate | No opinion | Adequate | Completely  adequate |
| 1. Communicating with patients |  |  |  |  |  |  |  |  |  |  |
| 2. Performing a thorough and complete examination |  |  |  |  |  |  |  |  |  |  |
| 3. Taking medical history |  |  |  |  |  |  |  |  |  |  |
| 4. Taking dental history |  |  |  |  |  |  |  |  |  |  |
| 5. Prescribing necessary laboratory tests |  |  |  |  |  |  |  |  |  |  |
| 6. Prescribing necessary intra-oral radiographs |  |  |  |  |  |  |  |  |  |  |
| 7. Interpreting intra-oral radiographs |  |  |  |  |  |  |  |  |  |  |
| 8. Prescribing necessary extra-oral radiographs |  |  |  |  |  |  |  |  |  |  |
| 9. Interpreting extra-oral radiographs |  |  |  |  |  |  |  |  |  |  |
| 10. Prescribing necessary drugs when needed |  |  |  |  |  |  |  |  |  |  |
| 11. Comprehensive treatment planning |  |  |  |  |  |  |  |  |  |  |
| 12. Diagnosing oral soft tissue lesions |  |  |  |  |  |  |  |  |  |  |
| 13. Restoring a relatively small cavity |  |  |  |  |  |  |  |  |  |  |
| 14. Restoring a big cavity involving more than two surfaces of the tooth |  |  |  |  |  |  |  |  |  |  |
| 15. Endodontic treatment of a single-root tooth |  |  |  |  |  |  |  |  |  |  |
| 16. Endodontic re-treatment of a single-root tooth |  |  |  |  |  |  |  |  |  |  |
| 17. Endodontic treatment of a multiple-root tooth |  |  |  |  |  |  |  |  |  |  |
| 18. Endodontic re-treatment of a multiple-root tooth |  |  |  |  |  |  |  |  |  |  |
| 19. Fabrication of removable complete denture |  |  |  |  |  |  |  |  |  |  |
| 20. Fabrication of removable partial denture |  |  |  |  |  |  |  |  |  |  |
| 21. Fabrication of a single crown |  |  |  |  |  |  |  |  |  |  |
| 22. Fabrication of a fixed partial prosthesis (bridge) |  |  |  |  |  |  |  |  |  |  |
| 23. Prosthetic laboratory technics and procedures |  |  |  |  |  |  |  |  |  |  |
| 24. Normal extraction of a single-root tooth |  |  |  |  |  |  |  |  |  |  |
| 25. Normal extraction of a multiple-root tooth except wisdom tooth |  |  |  |  |  |  |  |  |  |  |
| 26. Normal extraction of a wisdom tooth |  |  |  |  |  |  |  |  |  |  |
| 27. Simple surgical extraction of wisdom tooth |  |  |  |  |  |  |  |  |  |  |
| 28. Complicated surgical extraction of wisdom tooth |  |  |  |  |  |  |  |  |  |  |
| 29. Performing other intra-oral surgeries |  |  |  |  |  |  |  |  |  |  |
| 30. Basic treatments of periodontal diseases |  |  |  |  |  |  |  |  |  |  |
| 31. Performing periodontal surgeries |  |  |  |  |  |  |  |  |  |  |
| 32. Removable orthodontic treatments |  |  |  |  |  |  |  |  |  |  |
| 33. Restoring deciduous teeth |  |  |  |  |  |  |  |  |  |  |
| 34. Pulpotomy of a deciduous molar |  |  |  |  |  |  |  |  |  |  |
| 35. Pulpectomy of a deciduous molar |  |  |  |  |  |  |  |  |  |  |
| 36. Fabrication of stainless steel crown for a deciduous molar |  |  |  |  |  |  |  |  |  |  |
| 37. Fabrication of space-maintainer |  |  |  |  |  |  |  |  |  |  |
| 38. Preventive dentistry |  |  |  |  |  |  |  |  |  |  |
| 39. Community oral health |  |  |  |  |  |  |  |  |  |  |
| 40. Management of medical emergencies |  |  |  |  |  |  |  |  |  |  |
| 41. Management of dental emergencies |  |  |  |  |  |  |  |  |  |  |
| 42. Infection control |  |  |  |  |  |  |  |  |  |  |
| 43. Practice management |  |  |  |  |  |  |  |  |  |  |
| 44. Maintenance of dental equipment |  |  |  |  |  |  |  |  |  |  |
| 45. Professional behavior with other colleagues |  |  |  |  |  |  |  |  |  |  |
| 46. Performing a medical research |  |  |  |  |  |  |  |  |  |  |
| 47. Implementation of evidence-based dentistry principles |  |  |  |  |  |  |  |  |  |  |

Theoretical

B: In this section stick to only one option for each question.

What is your opinion in general about the adequacy of your dentistry program for achievement of required competencies for a general dentist?

1. Completely inadequate 2. Inadequate 3. No opinion 4. Adequate 5. Completely adequate

Section 3: Questions regarding the content density of the undergraduate dental curriculum

In this section there are 6 questions. Please show the level of your agreement with each of the following statements through selection of just one option.

A: The basic science phase is dense content-wise.

1. Strongly disagree 2. Disagree 3. No opinion 4. Agree 5. Strongly agree

B: It is better to extend the length of the basic science pahse.

1. Strongly disagree 2. Disagree 3. No opinion 4. Agree 5. Strongly agree

C: The pre-clinic phase is dense content-wise.

1. Strongly disagree 2. Disagree 3. No opinion 4. Agree 5. Strongly agree

D: It is better to extend the length of the pre-clinic phase.

1. Strongly disagree 2. Disagree 3. No opinion 4. Agree 5. Strongly agree

E: The clinic phase is dense content-wise.

1. Strongly disagree 2. Disagree 3. No opinion 4. Agree 5. Strongly agree

F: It is better to extend the length of the clinic phase.

1. Strongly disagree 2. Disagree 3. No opinion 4. Agree 5. Strongly agree

Section 4: Questions regarding the teaching methods of the undergraduate dental curriculum

In this section you are provided with 3 questions. Stick to only one option for each.

A: What do you think of the most commonly utilized teaching method (lecturing) in theoretical subjects?

1. Completely inappropriate 2. Inappropriate 3. No opinion 4. Appropriate 5. Completely appropriate

B: What do you think of the teaching methods utilized in the practical subjects of the preclinical 1. Completely inappropriate 2. Inappropriate 3. No opinion 4. Appropriate 5. Completely appropriate

C: What do you think of the teaching methods utilized in the practical subjects of clinical phase 1. Completely inappropriate 2. Inappropriate 3. No opinion 4. Appropriate 5. Completely appropriate
