# Supplementary material for: Evaluation of national dental curriculum in Iran using senior dental students’ feedback
Source: BMC Oral Health. 2023 Jan 26;23:45. doi: 10.1186/s12903-023-02757-x (PMC9876750; doi:10.1186/s12903-023-02757-x)
Supplement: Supplementary file 2 — Additional file 2: Appendix 2. Frequency distribution of the Iranian senior dental students (n=438) who believed in the adequacy of dentistry program to cover each of the curriculum-defined competencies, according to their gender. [file 12903_2023_2757_MOESM2_ESM.docx]

Appendix 2:

Frequency distribution of the Iranian senior dental students (n=438) who believed in the adequacy of dentistry program to cover each of the curriculum-defined competencies, according to their gender (theoretical domain)

|  |  | Gender |  |  |
| --- | --- | --- | --- | --- |
|  |  | Male  n (%) | Female  n (%) | P* |
| 1. Communicating with patients | 199 (45.7) | 80 (41.7) | 119 (49) | 0.13 |
| 2. Performing a thorough and complete examination | 288 (66.5) | 113 (59.2) | 175 (72.3) | 0.004 |
| 3. Taking medical history | 315 (73.4) | 130 (68.8) | 185 (77.1) | 0.05 |
| 4. Taking dental history | 325 (74.2) | 136 (72.3) | 189 (79.4) | 0.09 |
| 5. Prescribing necessary laboratory tests | 169 (39.2) | 71 (37.6) | 98 (40.5) | 0.54 |
| 6. Prescribing necessary intra-oral radiographs | 346 (82.0) | 151 (81.6) | 195 (82.3) | 0.86 |
| 7. Interpreting intra-oral radiographs | 300 (71.6) | 128 (69.6) | 172 (73.2) | 0.41 |
| 8. Prescribing necessary extra-oral radiographs | 213 (50.7) | 93 (50.3) | 120 (51.1) | 0.87 |
| 9. Interpreting extra-oral radiographs | 184 (44.2) | 87 (47.8) | 97 (41.5) | 0.20 |
| 10. Prescribing necessary drugs when needed | 158 (37.5) | 70 (38) | 88 (37.1) | 0.85 |
| 11. Comprehensive treatment planning | 185 (43.3) | 86 (45.7) | 99 (41.4) | 0.37 |
| 12. Diagnosing oral soft tissue lesions | 213 (50.6) | 87 (47.3) | 126 (53.0) | 0.23 |
| 13. Restoring a relatively small cavity | 355 (83.5) | 150 (79.4) | 205 (86.9) | 0.04 |
| 14. Restoring a big cavity involving more than two surfaces of the tooth | 311 (74.0) | 134 (72.8) | 177 (75.0) | 0.61 |
| 15. Endodontic treatment of a single-root tooth | 346 (82.0) | 151 (80.3) | 195 (83.3) | 0.42 |
| 16. Endodontic re-treatment of a single-root tooth | 163 (38.6) | 77 (41.2) | 86 (36.6) | 0.34 |
| 17. Endodontic treatment of a multiple-root tooth | 243(57.3) | 110 (57.9) | 133 (56.8) | 0.83 |
| 18. Endodontic re-treatment of a multiple-root tooth | 88 (21.0) | 45 (24.3) | 43 (18.4) | 0.14 |
| 19. Fabrication of removable complete denture | 291 (68.1) | 120 (63.8) | 171 (71.5) | 0.09 |
| 20. Fabrication of removable partial denture | 243 (57.2) | 95 (50.8) | 148 (62.2) | 0.02 |
| 21. Fabrication of a single crown | 278 (65.4) | 122 (64.9) | 156 (65.8) | 0.84 |
| 22. Fabrication of a fixed partial prosthesis (bridge) | 177 (41.8) | 79 (42.5) | 98 (41.4) | 0.82 |
| 23. Prosthetic laboratory technics and procedures | 195 (45.7) | 84 (44.7) | 111 (46.4) | 0.72 |
| 24. Normal extraction of a single-root tooth | 327 (77.1) | 141 (75.0) | 186 (78.8) | 0.35 |
| 25. Normal extraction of a multiple-root tooth except wisdom tooth | 286 (67.6) | 130 (69.9) | 156 (65.8) | 0.38 |
| 26. Normal extraction of a wisdom tooth | 223 (53.1) | 110 (58.8) | 113 (48.5) | 0.04 |
| 27. Simple surgical extraction of wisdom tooth | 152 (35.4) | 78 (41.1) | 74 (31.0) | 0.03 |
| 28. Complicated surgical extraction of wisdom tooth | 102 (23.8) | 49 (25.8) | 53 (22.2) | 0.38 |
| 29. Performing other intra-oral surgeries | 79 (18.5) | 34 (17.9) | 45 (19.0) | 0.77 |
| 30. Basic treatments of periodontal diseases | 246 (57.2) | 97 (50.8) | 149 (62.3) | 0.02 |
| 31. Performing periodontal surgeries | 111 (26.1) | 44 (23.3) | 67 (28.4) | 0.23 |
| 32. Removable orthodontic treatments | 217 (50.8) | 95 (50.3) | 122 (51.3) | 0.84 |
| 33. Restoring deciduous teeth | 349 (81.4) | 153 (80.1) | 196 (82.4) | 0.55 |
| 34. Pulpotomy of a deciduous molar | 340 (79.3) | 150 (78.9) | 190 (79.5) | 0.89 |
| 35. Pulpectomy of a deciduous molar | 314 (73.2) | 143 (74.9) | 171 (71.8) | 0.48 |
| 36. Fabrication of stainless steel crown for a deciduous molar | 266 (62.7) | 121 (63.7) | 145 (62.0) | 0.72 |
| 37. Fabrication of space-maintainer | 151 (35.3) | 83 (43.5) | 68 (28.7) | 0.001 |
| 38. Preventive dentistry | 210 (49.3) | 87 (45.8) | 123 (52.1) | 0.19 |
| 39. Community oral health | 184 (43.4) | 76 (40.0) | 108 (46.2) | 0.20 |
| 40. Management of medical emergencies | 143 (33.8) | 59 (31.4) | 84 (35.7) | 0.35 |
| 41. Management of dental emergencies | 163 (38.6) | 77 (40.7) | 86 (36.9) | 0.42 |
| 42. Infection control | 272 (63.8) | 108 (56.5) | 164 (69.8) | 0.005 |
| 43. Practice management | 80 (18.8) | 41 (21.5) | 39 (16.7) | 0.21 |
| 44. Maintenance of dental equipment | 77 (18.2) | 42 (22.3) | 35 (14.8) | 0.046 |
| 45. Professional behavior with other colleagues | 106 (31.4) | 50 (30.9) | 56 (31.8) | 0.85 |
| 46. Performing a medical research | 84 (24.7) | 44 (27.2) | 40 (22.5) | 0.32 |
| 47. Implementation of evidence-based dentistry principles | 70 (20.7) | 44 (27.2) | 26 (14.8) | 0.005 |

Chi-square test

Frequency distribution of the Iranian senior dental students (n=438) who believed in the adequacy of dentistry program to cover each of the curriculum-defined competencies, according to their gender (Practical domain)

|  |  | Gender |  |  |
| --- | --- | --- | --- | --- |
|  |  | Male  n (%) | Female  n (%) | P* |
| 1. Communicating with patients | 189 (44.7) | 77 (41.0) | 112 (47.7) | 0.17 |
| 2. Performing a thorough and complete examination | 250 (59.4) | 104 (55.9) | 146 (62.1) | 0.2 |
| 3. Taking medical history | 275 (65.8) | 119 (64.7) | 156 (66.7) | 0.67 |
| 4. Taking dental history | 283 (68.4) | 124 (67.0) | 159 (69.4) | 0.60 |
| 5. Prescribing necessary laboratory tests | 145 (34.8) | 63 (34.2) | 82 (35.2) | 0.84 |
| 6. Prescribing necessary intra-oral radiographs | 318 (77.4) | 137 (74.9) | 181 (79.4) | 0.28 |
| 7. Interpreting intra-oral radiographs | 273 (66.4) | 121 (66.9) | 152 (66.1) | 0.871 |
| 8. Prescribing necessary extra-oral radiographs | 185 (44.5) | 79 (43.2) | 106 (45.5) | 0.64 |
| 9. Interpreting extra-oral radiographs | 158 (38.9) | 71 (40.3) | 87 (37.8) | 0.61 |
| 10. Prescribing necessary drugs when needed | 111 (27.2) | 49 (27.1) | 62 (27.3) | 0.96 |
| 11. Comprehensive treatment planning | 165 (40.1) | 80 (44.4) | 85 (36.8) | 0.12 |
| 12. Diagnosing oral soft tissue lesions | 145 (35.4) | 69 (37.9) | 76 (33.3) | 0.34 |
| 13. Restoring a relatively small cavity | 327 (79.6) | 138 (75.4) | 189 (82.9) | 0.06 |
| 14. Restoring a big cavity involving more than two surfaces of the tooth | 293 (71.5) | 121 (66.5) | 172 (75.4) | 0.046 |
| 15. Endodontic treatment of a single-root tooth | 321 (77.5) | 140 (76.5) | 181 (78.4) | 0.65 |
| 16. Endodontic re-treatment of a single-root tooth | 134 (32.4) | 60 (32.6) | 74 (32.2) | 0.93 |
| 17. Endodontic treatment of a multiple-root tooth | 189 (45.7) | 86 (46.7) | 103 (44.8) | 0.69 |
| 18. Endodontic re-treatment of a multiple-root tooth | 71 (17.3) | 38 (20.7) | 33 (14.5) | 0.10 |
| 19. Fabrication of removable complete denture | 264 (63.3) | 101 (55.2) | 163 (69.7) | 0.002 |
| 20. Fabrication of removable partial denture | 205 (48.7) | 83 (44.9) | 122 (51.7) | 0.16 |
| 21. Fabrication of a single crown | 239 (57.3) | 104 (57.1) | 135 (57.4) | 0.95 |
| 22. Fabrication of a fixed partial prosthesis (bridge) | 152 (36.5) | 67 (36.4) | 85 (36.6) | 0.96 |
| 23. Prosthetic laboratory technics and procedures | 171 (41.1) | 71 (38.6) | 100 (43.1) | 0.35 |
| 24. Normal extraction of a single-root tooth | 301 (73.1) | 127 (70.2) | 174 (75.3) | 0.24 |
| 25. Normal extraction of a multiple-root tooth except wisdom tooth | 235 (56.8) | 106 (58.6) | 129 (55.4) | 0.52 |
| 26. Normal extraction of a wisdom tooth | 207 (49.6) | 97 (53.0) | 110 (47.0) | 0.224 |
| 27. Simple surgical extraction of wisdom tooth | 114 (27.2) | 62 (33.7) | 52 (22.1) | 0.008 |
| 28. Complicated surgical extraction of wisdom tooth | 68 (16.3) | 35 (19.1) | 33 (14.1) | 0.17 |
| 29. Performing other intra-oral surgeries | 57 (13.8) | 33 (17.9) | 24 (10.4) | 0.03 |
| 30. Basic treatments of periodontal diseases | 219 (52.5) | 85 (46.2) | 134 (57.5) | 0.02 |
| 31. Performing periodontal surgeries | 74 (17.8) | 35 (19.1) | 39 (16.7) | 0.53 |
| 32. Removable orthodontic treatments | 188 (45.1) | 79 (43.4) | 109 (46.4) | 0.55 |
| 33. Restoring deciduous teeth | 325 (77.8) | 136 (73.9) | 189 (80.8) | 0.09 |
| 34. Pulpotomy of a deciduous molar | 295 (70.6) | 127 (69.0) | 168 (71.8) | 0.54 |
| 35. Pulpectomy of a deciduous molar | 268 (64.0) | 115 (62.5) | 153 (65.1) | 0.58 |
| 36. Fabrication of stainless steel crown for a deciduous molar | 206 (49.8) | 89 (48.6) | 117 (50.6) | 0.68 |
| 37. Fabrication of space-maintainer | 89 (21.3) | 50 (27.0) | 39 (16.7) | 0.01 |
| 38. Preventive dentistry | 187 (45.2) | 76 (41.1) | 111 (48.5) | 0.13 |
| 39. Community oral health | 170 (41.0) | 66 (35.7) | 104 (45.2) | 0.049 |
| 40. Management of medical emergencies | 72 (17.5) | 29 (16.1) | 43 (18.6) | 0.51 |
| 41. Management of dental emergencies | 103 (25.1) | 50 (27.3) | 53 (23.3) | 0.36 |
| 42. Infection control | 234 (56.7) | 90 (49.5) | 144 (62.3) | 0.009 |
| 43. Practice management | 67 (16.2) | 37 (20.3) | 30 (12.9) | 0.04 |
| 44. Maintenance of dental equipment | 62 (14.9) | 35 (19.1) | 27 (11.6) | 0.03 |
| 45. Professional behavior with other colleagues | 93 (28.3) | 44 (28.6) | 49 (28.0) | 0.91 |
| 46. Performing a medical research | 68 (20.5) | 35 (22.3) | 33 (19.0) | 0.45 |
| 47. Implementation of evidence-based dentistry principles | 64 (19.5) | 42 (26.9) | 22 (12.8) | 0.001 |

Chi-square test
