# Supplementary material for: Evaluation of national dental curriculum in Iran using senior dental students’ feedback
Source: BMC Oral Health. 2023 Jan 26;23:45. doi: 10.1186/s12903-023-02757-x (PMC9876750; doi:10.1186/s12903-023-02757-x)
Supplement: Supplementary file 3 — Additional file 3: Appendix 3. Frequency distribution of the responses by Iranian senior dental students (n=438) to the questions regarding adequacy of their dentistry program to cover each of the curriculum-defined competencies, appropriateness of the teaching methods used in the three phases of the national dental curriculum, intensity of the national dental curriculum in its three phases, and agreement with the necessity of the extension of the three phases. [file 12903_2023_2757_MOESM3_ESM.docx]

Appendix 3:

Table 1S3. Frequency distribution of the responses by Iranian senior dental students (n=438) to the questions regarding adequacy of their dentistry program to cover each of the curriculum-defined competencies (theoretical domain)

|  |  |  |  |
| --- | --- | --- | --- |
|  | Completely inadequate or inadequate  (%) | No opinion  (%) | Adequate or completely adequate  (%) |
| 1. Communicating with patients | 173 (39.8) | 63 (14.4) | 199 (45.7) |
| 2. Performing a thorough and complete examination | 88 (20.3) | 57 (13.0) | 288 (66.5) |
| 3. Taking medical history | 76 (17.7) | 38 (8.9) | 315 (73.4) |
| 4. Taking dental history | 54 (12.7) | 47 (11.0) | 325 (76.3) |
| 5. Prescribing necessary laboratory tests | 203 (47.1) | 59 (13.7) | 169 (39.2) |
| 6. Prescribing necessary intra-oral radiographs | 37 (8.8) | 39 (9.2) | 346 (82.0) |
| 7. Interpreting intra-oral radiographs | 71 (16.9) | 48 (11.5) | 300 (71.6) |
| 8. Prescribing necessary extra-oral radiographs | 125 (29.8) | 82 (19.5) | 213 (50.7) |
| 9. Interpreting extra-oral radiographs | 153 (36.8) | 79 (19.0) | 184 (44.2) |
| 10. Prescribing necessary drugs when needed | 201 (47.7) | 62 (14.7) | 158 (37.5) |
| 11. Comprehensive treatment planning | 163 (38.2) | 79 (18.5) | 185 (43.3) |
| 12. Diagnosing oral soft tissue lesions | 151 (35.9) | 57 (13.5) | 213 (50.6) |
| 13. Restoring a relatively small cavity | 41 (9.6) | 29 (6.8) | 355 (83.5) |
| 14. Restoring a big cavity involving more than two surfaces of the tooth | 63 (15.0) | 46 (11.0) | 311 (74.0) |
| 15. Endodontic treatment of a single-root tooth | 39 (9.2) | 37 (8.8) | 346 (82.0) |
| 16. Endodontic re-treatment of a single-root tooth | 198 (46.8) | 61 (14.4) | 163 (38.5) |
| 17. Endodontic treatment of a multiple-root tooth | 121 (28.5) | 60 (14.2) | 243 (57.3) |
| 18. Endodontic re-treatment of a multiple-root tooth | 279 (66.6) | 52 (12.4) | 88 (21.0) |
| 19. Fabrication of removable complete denture | 86 (20.1) | 50 (11.7) | 291 (68.1) |
| 20. Fabrication of removable partial denture | 129 (30.4) | 53 (12.5) | 243 (57.2) |
| 21. Fabrication of a single crown | 94 (22.1) | 53 (12.5) | 278 (65.4) |
| 22. Fabrication of a fixed partial prosthesis (bridge) | 173 (40.9) | 73 (17.3) | 177 (41.8) |
| 23. Prosthetic laboratory technics and procedures | 154 (36.1) | 78 (18.3) | 195 (45.7) |
| 24. Normal extraction of a single-root tooth | 61 (14.4) | 36 (8.5) | 327 (77.1) |
| 25. Normal extraction of a multiple-root tooth except wisdom tooth | 90 (21.3) | 47 (11.1) | 286 (67.6) |
| 26. Normal extraction of a wisdom tooth | 139 (33.1) | 58 (13.8) | 223 (53.1) |
| 27. Simple surgical extraction of wisdom tooth | 226 (52.7) | 51 (11.9) | 152 (35.4) |
| 28. Complicated surgical extraction of wisdom tooth | 280 (65.3) | 47 (11.0) | 102 (23.8) |
| 29. Performing other intra-oral surgeries | 299 (70.0) | 49 (11.5) | 79 (18.5) |
| 30. Basic treatments of periodontal diseases | 126 (29.3) | 58 (13.5) | 246 (57.2) |
| 31. Performing periodontal surgeries | 260 (61.2) | 54 (12.7) | 111 (26.1) |
| 32. Removable orthodontic treatments | 145 (34.0) | 65 (15.2) | 217 (50.8) |
| 33. Restoring deciduous teeth | 43 (10.0) | 37 (8.6) | 349 (81.4) |
| 34. Pulpotomy of a deciduous molar | 47 (11.0) | 42 (9.8) | 340 (79.3) |
| 35. Pulpectomy of a deciduous molar | 70 (16.3) | 45 (10.5) | 314 (73.2) |
| 36. Fabrication of stainless steel crown for a deciduous molar | 108 (25.5) | 50 (11.8) | 266 (62.7) |
| 37. Fabrication of space-maintainer | 211 (49.3) | 66 (15.4) | 151 (35.3) |
| 38. Preventive dentistry | 131 (30.8) | 85 (20.0) | 210 (49.3) |
| 39. Community oral health | 136 (32.1) | 104 (24.5) | 184 (43.4) |
| 40. Management of medical emergencies | 219 (51.7) | 61 (14.4) | 143 (33.7) |
| 41. Management of dental emergencies | 201 (47.6) | 58 (13.7) | 163 (38.6) |
| 42. Infection control | 97 (22.8) | 57 (13.4) | 272 (63.8) |
| 43. Practice management | 280 (65.9) | 65 (15.3) | 80 (18.8) |
| 44. Maintenance of dental equipment | 288 (67.9) | 59 (13.9) | 77 (18.2) |
| 45. Professional behavior with other^*^ colleagues | 165 (48.8) | 67 (19.8) | 106 (31.4) |
| 46. Performing a medical research^*^ | 205 (60.3) | 51 (15.0) | 84 (24.7) |
| 47. Implementation of evidence-based dentistry principles^*^ | 193 (57.1) | 75 (22.2) | 70 (20.7) |

* These questions, which were at the end of the list in the distributed questionnaire, left un-answered by the students of one of the dental schools. Regarding other questions, there were up to 22 (6%) non-responses.

Table 2S3. Frequency distribution of the responses by Iranian senior dental students (n=438) to the questions regarding adequacy of their dentistry program to cover each of the curriculum-defined competencies (practical domain)

|  |  |  |  |
| --- | --- | --- | --- |
|  | Completely inadequate or inadequate  (%) | No opinion  (%) | Adequate or completely adequate  (%) |
| 1. Communicating with patients | 178 (42.1) | 56 (13.2) | 189 (44.7) |
| 2. Performing a thorough and complete examination | 116 (27.6) | 55 (13.1) | 250 (59.4) |
| 3. Taking medical history | 101 (24.2) | 42 (10.0) | 275 (65.8) |
| 4. Taking dental history | 81 (19.6) | 50 (12.1) | 283 (68.4) |
| 5. Prescribing necessary laboratory tests | 213 (51.1) | 59 (14.1) | 145 (34.8) |
| 6. Prescribing necessary intra-oral radiographs | 53 (12.9) | 40 (9.7) | 318 (77.4) |
| 7. Interpreting intra-oral radiographs | 94 (22.9) | 44 (10.7) | 273 (66.4) |
| 8. Prescribing necessary extra-oral radiographs | 157 (37.7) | 74 (17.8) | 185 (44.5) |
| 9. Interpreting extra-oral radiographs | 178 (43.8) | 70 (17.2) | 158 (38.9) |
| 10. Prescribing necessary drugs when needed | 235 (57.6) | 62 (15.2) | 111 (27.2) |
| 11. Comprehensive treatment planning | 170 (41.3) | 76 (18.4) | 165 (40.0) |
| 12. Diagnosing oral soft tissue lesions | 203 (49.5) | 62 (15.1) | 145 (35.4) |
| 13. Restoring a relatively small cavity | 43 (10.5) | 41 (10.0) | 327 (79.6) |
| 14. Restoring a big cavity involving more than two surfaces of the tooth | 78 (19.0) | 39 (9.5) | 293 (71.3) |
| 15. Endodontic treatment of a single-root tooth | 60 (14.5) | 33 (8.0) | 321 (77.5) |
| 16. Endodontic re-treatment of a single-root tooth | 216 (52.2) | 64 (15.5) | 134 (32.4) |
| 17. Endodontic treatment of a multiple-root tooth | 167 (40.3) | 58 (14.0) | 189 (45.7) |
| 18. Endodontic re-treatment of a multiple-root tooth | 283 (68.7) | 57 (13.8) | 71 (17.2) |
| 19. Fabrication of removable complete denture | 94 (22.5) | 59 (14.1) | 264 (63.3) |
| 20. Fabrication of removable partial denture | 136 (32.3) | 80 (19.0) | 205 (48.7) |
| 21. Fabrication of a single crown | 120 28.8) | 58 (13.9) | 239 (57.3) |
| 22. Fabrication of a fixed partial prosthesis (bridge) | 204 (49.0) | 60 (14.4) | 152 (36.5) |
| 23. Prosthetic laboratory technics and procedures | 176 (42.3) | 69 (16.6) | 171 (41.1) |
| 24. Normal extraction of a single-root tooth | 70 (17.0) | 41 (10.0) | 301 (73.1) |
| 25. Normal extraction of a multiple-root tooth except wisdom tooth | 121 (29.2) | 58 (14.0) | 235 (56.8) |
| 26. Normal extraction of a wisdom tooth | 162 (38.8) | 48 (11.5) | 207 (49.6) |
| 27. Simple surgical extraction of wisdom tooth | 259 (61.7) | 46 (11.0) | 114 (27.1) |
| 28. Complicated surgical extraction of wisdom tooth | 305 (73.0) | 44 (10.5) | 68 (16.3) |
| 29. Performing other intra-oral surgeries | 317 (76.4) | 40 (9.6) | 57 (13.7) |
| 30. Basic treatments of periodontal diseases | 144 (34.5) | 54 (12.9) | 219 (52.5) |
| 31. Performing periodontal surgeries | 298 (71.6) | 44 (10.6) | 74 (17.8) |
| 32. Removable orthodontic treatments | 171 (41.0) | 58 (13.9) | 188 (45.1) |
| 33. Restoring deciduous teeth | 50 (12.0) | 43 (10.3) | 325 (77.8) |
| 34. Pulpotomy of a deciduous molar | 64 (15.3) | 59 (14.1) | 295 (70.6) |
| 35. Pulpectomy of a deciduous molar | 92 (16.3) | 59 (14.1) | 314 (73.2) |
| 36. Fabrication of stainless steel crown for a deciduous molar | 108 (25.5) | 50 (11.8) | 268 (64.0) |
| 37. Fabrication of space-maintainer | 269 (64.4) | 60 (14.4) | 89 (21.3) |
| 38. Preventive dentistry | 157 (37.9) | 70 (16.9) | 187 (45.2) |
| 39. Community oral health | 144 (34.7) | 101 (24.3) | 170 (41.0) |
| 40. Management of medical emergencies | 283 (68.9) | 56 (13.6) | 72 (17.5) |
| 41. Management of dental emergencies | 248 (60.5) | 59 (14.4) | 103 (25.1) |
| 42. Infection control | 123 (29.8) | 56 (13.6) | 234 (56.7) |
| 43. Practice management | 280 (67.6) | 67 (16.2) | 67 (16.2) |
| 44. Maintenance of dental equipment | 297 (71.6) | 56 (13.5) | 62 (14.9) |
| 45. Professional behavior with other colleagues^*^ | 173 (52.6) | 63 (19.1) | 93 (28.3) |
| 46. Performing a medical research^*^ | 215 (65.0) | 48 (14.5) | 68 (20.5) |
| 47. Implementation of evidence-based dentistry principles^*^ | 183 (57.1) | 81 (24.7) | 64 (19.5) |

* These questions, which were at the end of the list in the distributed questionnaire, left un-answered by the students of one of the dental schools. Regarding other questions, there were up to 30 (7%) non-responses.

Table 3S3. Frequency distribution of the responses by Iranian senior dental students (n=438) to the questions regarding appropriateness of the teaching methods used in the three phases of the national dental curriculum

|  | Completely inappropriate or inappropriate  (%) | No opinion  (%) | Appropriate or completely appropriate  (%) |
| --- | --- | --- | --- |
|  |  |  |  |
| Theoretical courses | 223 (51.6) | 91 (21.1) | 118 (27.3) |
| Practical courses in the pre-clinical phase | 252 (58.1) | 54 (12.4) | 128 (29.5) |
| Practical courses in the clinical phase | 258 (59.3) | 68 (15.6) | 109 (25.1) |

Table 4S3. Frequency distribution of the responses by Iranian senior dental students (n=438) to the questions regarding intensity of the national dental curriculum in its three phases

|  | Completely disagree or disagree  (%) | No opinion  (%) | Agree or completely agree  (%) |
| --- | --- | --- | --- |
|  |  |  |  |
| Basic sciences phase | 123 (28.2) | 75 (17.2) | 238 (54.6) |
| Pre-clinical phase | 154 (35.5) | 88 (20.3) | 192 (44.2) |
| Clinical phase | 167 (38.4) | 84 (19.3) | 184 (42.3) |

Table 5S3. Frequency distribution of the responses by Iranian senior dental students (n=438) to the questions regarding agreement with the necessity of the extension of the three phases of national dental curriculum

|  | Completely disagree or disagree  (%) | No opinion  (%) | Agree or completely agree  (%) |
| --- | --- | --- | --- |
|  |  |  |  |
| Basic sciences phase | 366 (83.9) | 37 (8.5) | 33 (7.6) |
| Pre-clinical phase | 269 (61.8) | 58 (13.3) | 108 (24.8) |
| Clinical phase | 119 (27.3) | 51 (11.7) | 266 (61.0) |
